# The Effects of Medium Spiny Neuron Morphologcial Changes on Basal Ganglia Network under External Electric Field: A Computational Modeling Study

**DOI:** 10.3389/fncom.2017.00091

**Published:** 2017-10-26

**Authors:** Xiaohan Zhang, Shenquan Liu, Feibiao Zhan, Jing Wang, Xiaofang Jiang

**Affiliations:** ^1^Department of Mathematics, South China University of Technology, Guangzhou, China; ^2^Department of Mathematics and Science, Henan Institute of Science and Technology, Xinxiang, China

**Keywords:** Parkinson's disease, BG network, spine loss, dendrites degeneration, computational model, medium spiny neuron, electromagnetic induction

## Abstract

The damage of dopaminergic neurons that innervate the striatum has been considered to be the proximate cause of Parkinson's disease (PD). In the dopamine-denervated state, the loss of dendritic spines and the decrease of dendritic length may prevent medium spiny neuron (MSN) from receiving too much excitatory stimuli from the cortex, thereby reducing the symptom of Parkinson's disease. However, the reduction in dendritic spine density obtained by different experiments is significantly different. We developed a biological-based network computational model to quantify the effect of dendritic spine loss and dendrites tree degeneration on basal ganglia (BG) signal regulation. Through the introduction of error index (EI), which was used to measure the attenuation of the signal, we explored the amount of dendritic spine loss and dendritic trees degradation required to restore the normal regulatory function of the network, and found that there were two ranges of dendritic spine loss that could reduce EI to normal levels in the case of dopamine at a certain level, this was also true for dendritic trees. However, although these effects were the same, the mechanisms of these two cases were significant difference. Using the method of phase diagram analysis, we gained insight into the mechanism of signal degradation. Furthermore, we explored the role of cortex in MSN morphology changes dopamine depletion-induced and found that proper adjustments to cortical activity do stop the loss in dendritic spines induced by dopamine depleted. These results suggested that modifying cortical drive onto MSN might provide a new idea on clinical therapeutic strategies for Parkinson's disease.

## 1. Introduction

There is no doubt that the main cause of Parkinson's disease is the reduction of dopamine concentration in the striatum (Fahn, 2003). A prevailing view about the importance of dopamine is to regulate the formation of dendritic spines and maintain the density of dendritic spines (Robinson and Kolb, 1999). Previous postmortem studies of PD have reported that a substantial loss in MSN dendritic segments and total dendritic length just happened in the putamen (McNeill et al., 1988). However, it was in the caudate nucleus that the density of dendritic spines and the size of the dendritic trees were also significantly reduced (Stephens et al., 2005). Similar morphological changes have also occurred in the Parkinson's syndrome animal model (Day et al., 2006). These changes in dendritic structure protect MSNs against being exposed to excessive glutamate excitatory stimuli (Deutch, 2006), furthermore, all these morphological changes are persistent, even the use of levodopa does not prevent these changes, nor it can reverse these changes (Zajamilatovic et al., 2005). These conclusions may explain the benefits of levodopa tend to reduce in late-stage Parkinson's disease (Follett et al., 2010; Tomlinson et al., 2010). However, some experimental studies have shown that there were no change in a relative distribution of thin and stubby spines or loss of dendritic spines in the culture where the cortex was stripped (Deutch, 2006), suggesting that the excitatory input of corticostriatal glutamatergic could play an important role in the morphology changes of MSN neurons induced by dopamine damage. Therefore, we first discussed the electrical activity of modified cortical neuron under external electric field separately. Dynamical response of a modified Hodgkin–Huxley neuron model and phase transition in modes were detected under electromagnetic induction and additive phase noise (Wu et al., 2017). Ma et al. discussed the synchronization behaviors of coupled Hindmarsh–Rose neuronal model under electromagnetic radiation (Ma et al., 2017).

In different experimental studies, the reductions of dendritic spines density and dendritic length are not the same. The density of dendritic spines showed a non-significant reduction by about 19% in animals studies (Ingham et al., 1998), and a much lower value was observed in Yelnik et al. (1991). The spines density decreased by about 27% in Stephens et al. (2005), which was smaller than the value described in the paper (Graveland et al., 1985). Day et al. found a significant reduction in dendritic spines of 50% in MSN. In PD patients, there was about a 21% decrease in the total dendritic length (Stephens et al., 2005) compared with a significant 40% decrease (McNeill et al., 1988) in the same nucleus.

**Figure 1:**
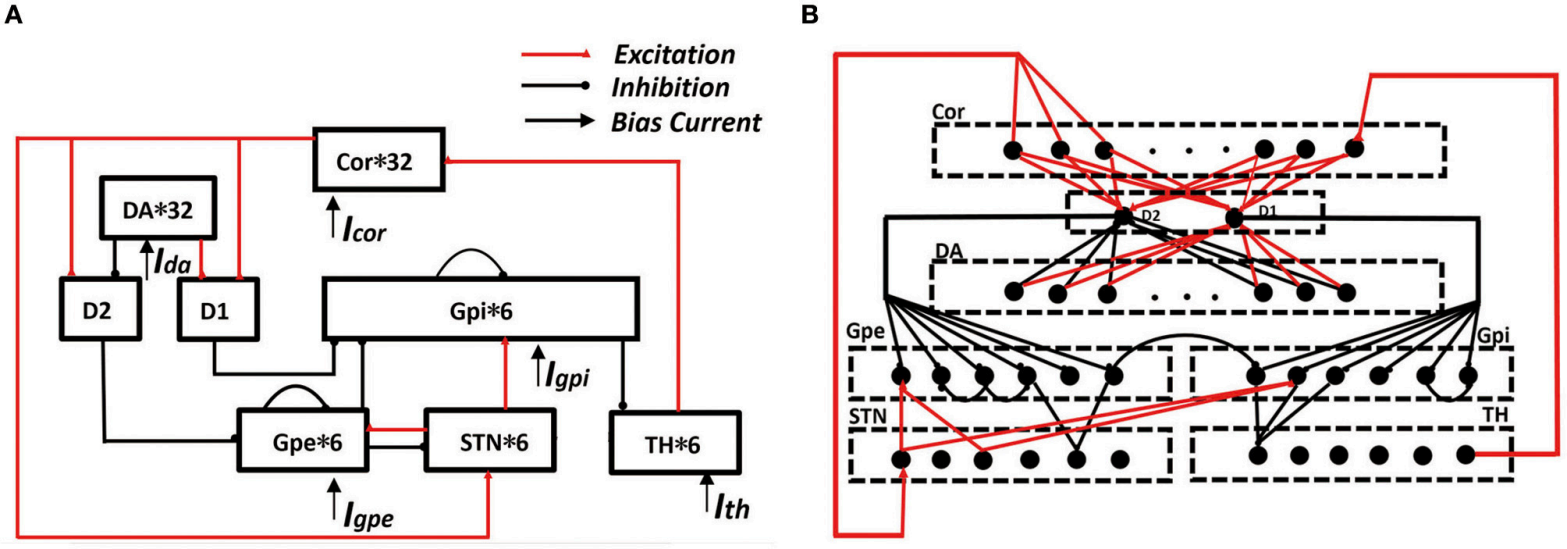
Framework of cortical-basal ganglia-thalamic circuitry (BG model). **(A)** The connection diagram of the model within the network. **(B)** A detailed description of synaptic connections in network model. In the cortex, six of the randomly selected neurons receives excitatory input from TH. D1 accepts excitatory input from each cortex and also accepts excitatory input from each DA. D2 accepts excitatory input from each cortex and the inhibitory input from each DA. Each STN neuron receives excitatory input from three cortices and receives two GPe inhibitory inputs. Each GPe receives two adjacent GPe suppressions, and also accepts the excitatory input from D2 and the excitatory input of STN. Each GPi neuron receives inhibitory input from D1 and inhibitory input from two randomly selected GPe neurons. At the same time, it also accepts two STN excitatory stimuli and the suppression of a neighboring neuron. Each TH neuron receives three GPi inhibitory inputs.

However, there is no current computational model to quantify the response of dendritic spine loss and dendrites tree degradation to different degree of the damage of dopaminergic neurons. Therefore, we elected to modify the the basal ganglia-thalamic network model developed by Rubin and Terman (RT model; Terman et al., 2002; Rubin and Terman, 2004). Different from most of the previous models, the models we constructed specifically included the medium spiny neurons (MSNs) with a multi-compartment stylized morphology, which was developed based on the anatomical experiments of animals and the electrophysiological parameters of different compartment. Since it was very similar to the neuronal cells of the actual mice, it could almost actually simulate the loss of dendritic spines and the degradation of dendrites tree. With this model in place we set out to explore the effects of the loss of spines on thalamus discharge rhythm and identify a neuronal activity metric (error index) which closely correlated with basal ganglia normal function. The simulation in this paper reproduced the previous experimental results, supporting the hypothesis that dopamine gates glutamatergic drive onto MSN and the compensatory mechanisms works both by reducing the expression of glutamate receptors. The results suggested that the EI indicators could be minimized in a few areas where the dendrites degenerated or the spines lost, but the mechanism that caused EI reduction was very different. Moreover, we used the BG network (Figure 1) to study the role of cortex in MSN morphology changes dopamine depletion-induced, and found that appropriate adjustment of the cortical stimulus intensity and stimulus frequency could restore the basal ganglia to normal function, thus preventing or slowing the dystrophy changes in MSN morphology. This may be effective in preventing or slowing the loss of responsiveness to L-dopa medical treatment.

## 2. Models and methods

In RT model, there were four neuronal structures: these are the thalamus neuron (TH), subthalamic nucleus (STN), globus pallidus externa (GPe), and globus pallidus interna (GPi). The thalamus accepts inhibition input from GPi and excitatory input. GPi and GPe both accepts excitatory input from STN. Moreover, GPi accepts inhibition input from GPe, and there is interpallidal inhibition among the GPe neurons. Finally, STN accepts inhibition input from GPe as well.

The model applied in our simulations consisted of eight types of neuronal structures. There were the pyramidal neuron (PY), dopamine d1 receptor (D1), dopamine d2 receptor (D2), dopaminergic neuron (DA). The other four neurons (TH, STN, GPe, GPi) are derived from the RT model. Here, D1 represent the MSNs that works on the direct pathway and D2 represent the MSNs that works on the indirect pathway. The cortical-MSN and DA-MSN connectivity configurations were all to one connections, and all other connectivity abided by a deterministic pattern based on RT model. Besides, the model included interpallidal inhibition among the GPi neurons. These neurons comprised a functional network by model synapses connections. The transition from the normal state to the PD state was achieved by removing the dopaminergic neurons. When dopamine neurons were reduced by more than 50%, this state was defined as PD state. The result was a reduction in the excitement of the direct pathway to TH, as well as the inhibitory effect of the indirect pathway. All the model equations and some corresponding parameters are detailed in Supplementary Material. The construction of the network was implemented by means of NEURON software and ran with time step of 0.025 ms. Moreover, we processed the data with Python and handled the image with origins 9.0.

### 2.1. Evaluation of model performance

BG network performance was assessed by verifying the attenuation of the signal. We used EI, described by Rubin and Terman (2004), to measure the normality of thalamic throughput. The dieaway of the signal was measured by the number of peaks of TH discharge over a period of time. It has been clarified that the denaturation of dopaminergic neurons in the substantia nigra and the decrease in striatal dopamine levels were the initiation events that induced Parkinson's disease. The reduction of dopamine caused its regulation about basal ganglia to be uncoordinated, resulting in the electrical signal delivered to the effector, this referred to TH, was lost or incomplete. In this paper, the peak type was divided into three kinds: lost peak, incomplete peak, normal peak. We took the rest state as a lost peak; 10–90% of the maximum peak as an incomplete peak; 90–100% of the maximum peak as a normal peak. EI was identified as the number of incomplete peak divided by the number of normal peak. For each state tested, the duration of the simulation was 16 s.

### 2.2. Each cell in the model

Here, in addition to MSN is a multi-compartment model, all others neurons are single-compartment conductance-based models applied in previous theoretical analysis of the network model.

#### 2.2.1. Thalamic neurons

We made a slight change to the TH neurons in the RT model. Constant current (*I*_*appth*_) that represented excitatory input from other brain regions (Such as the cerebellum) was applied to the model to replace sensorimotor cortical (SMC) input in the original model. Membrane potential (υ_*th*_) of the TH cell was determined by the current balance equation:

(1) $$\begin{array}{l} C_{m}\upsilon_{th}\prime =-I_{L}-I_{Na}-I_{K}-I_{T}-I_{gpi\to th}+I_{appth} \end{array}$$

The details of the ion current were described in RT in published reports (Rubin and Terman, 2004). We used *I*_*gpi*→*th*_ to denote the inhibitory input coming from GPi, which represented the input of hyperpolarizing current. Moreover, in the absence of external stimuli, TH could not spontaneously discharge.

#### 2.2.2. STN neuron

The STN neurons model were derived from previous studies and extended the previous model (Terman et al., 2002). When there was no external current input, STN presented a low frequency spontaneous discharge and showed a high frequency discharge with full excitatory stimulation. Membrane potential (υ_*stn*_) of the STN cell was determined by the current balance equation:

(2) $$\begin{array}{l} C_{m}\upsilon_{stn}\prime =-I_{L}-I_{Na}-I_{K}-I_{T}-I_{Ca}-I_{ahp}-I_{gpe\to stn}\\ \;-I_{cor\to stn} \end{array}$$

The ionic current was similar to the above TH neurons. *I*_*gpe*→*stn*_ denoted the excitatory synaptic input from GPe. We increased the excitatory synaptic input from the cortex to STN, denoted as *I*_*cor*→*stn*_.

#### 2.2.3. GP neuron

The GPe and GPi neurons model were derived from a previous model (Rosa et al., 2012). We used *I*_*d*2 → *gpe*_ to represent the striatum's inhibitory input to GPe instead of using the bais current. The constant bias current (*I*_*appgpe*_) applied to GPe was to represent synaptic input from other brain regions which were included in the model. Membrane potential (υ_*gpe*_) of the GPe cell was determined by the current balance equation:

(3) $$\begin{array}{l} C_{m}\upsilon_{gpe}\prime =-I_{L}-I_{Na}-I_{K}-I_{T}-I_{Ca}-I_{ahp}-I_{d2\to gpe}\\ \;-I_{gpe\to gpe}-I_{stn\to gpe}-I_{appgpe} \end{array}$$

GPi was the main output nucleus of the basal ganglia, we used the following current balance equation to calculate the GPi membrane potential (υ_*gpi*_):

(4) $$\begin{array}{l} C_{m}\upsilon_{gpi}\prime =-I_{L}-I_{Na}-I_{K}-I_{T}-I_{Ca}-I_{ahp}-I_{d1\to gpi}\\ \;-I_{gpe\to gpi}-I_{gpi\to gpi}-I_{stn\to gpi}+I_{appgpi} \end{array}$$

The ion currents of GPe and GPi were the same as described above. *I*_*d*1 → *gpi*_, *I*_*gpe*/*gpi*→*gpi*_, and *I*_*stn*→*gpi*_ are the synaptic inputs from D1, GP, and STN, respectively. The constant bias current (*I*_*appgpi*_) applied to GPi was to represent synaptic input from other brain regions analogous to GPe. GPe and GPi could not spontaneously discharge in a resting state.

#### 2.2.4. Pyramidal neuron

Pyramidal cells were the main projection neurons of the cerebral cortex. The model described here was developed by a published report (Pospischil et al., 2008). Membrane potential (υ_*cor*_) of the pyramidal cell was determined by the current balance equation:

(5) $$\begin{array}{l} C_{m}\upsilon_{cor}\prime =-I_{leak}-I_{Na}-I_{Kd}-I_{M}-I_{L}-I_{th\to cor}+I_{appcor} \end{array}$$

Where *I*_*leak*_ is the leak current, *I*_*Kd*_ and *I*_*Na*_ are the potassium and sodium currents, respectively, and responsible for generating action potential. *I*_*L*_ is the high-threshold calcium current. *I*_*M*_, responsible for the adaptation of firing rate and the after hyperpolarization of cortical pyramidal cells, is the voltage-dependent slow potassium current. *I*_*appcor*_ indicates external excitatory stimulation and *I*_*th*→*cor*_ represents the feedback of the thalamus to the brain.

#### 2.2.5. Dopaminergic neurons

Dopaminergic neurons are involved in motivation and the control of movement, and neurodegeneration of dopaminergic neurons in the substantia nigra has been implicated in progressive neurodegenerative disease such as Parkinson's disease (Bernheimer et al., 1973). Moreover, the amount of dopamine released in the projection areas are determined by the discharge pattern in dopaminergic neurons (Gonon, 1988). In this paper, the model was based on a multi-compartment model (Komendantov et al., 2004; Canavier and Landry, 2006). In the original model, the neuron's topological structure consisted of a cell body, four trunks and eight secondary dendrites. Here we considered the single-compartment only with a soma. Membrane potential (υ_*DA*_) of the dopaminergic cell was determined by the current balance equation:

(6) $$\begin{array}{l} C_{m}\upsilon_{DA}\prime =-I_{Na}-I_{A}-I_{KDR}-I_{K,SK}-I_{NaP}-I_{Ca,T}-I_{Ca,L}\\ \;-I_{Ca,N}-I_{Ca,P}-I_{L,Na}-I_{L,K}-I_{L,Ca}+I_{appDA} \end{array}$$

Where *I*_*Na*_, *I*_*KDR*_, and *I*_*A*_ denote fast sodium current, a delayed rectifying potassium current and potassium A channels, respectively. *I*_*K,SK*_ represents calcium dependent potassium channel which activates the SK channel. *I*_*NaP*_, and *I*_*Ca,P*_ are sodium pump and calcium pump. *I*_*L,Na*_, *I*_*L,K*_, *I*_*L,Ca*_ are the leak current. The soma also consists of voltage-activated T-type calcium currents, N-type calcium currents, and L-type calcium currents which are represented by *I*_*Ca,T*_, *I*_*Ca,N*_, and *I*_*Ca,L*_, respectively. *I*_*appDA*_ indicates external excitatory stimulation.

#### 2.2.6. Medium spiny neuron

The striatum is made up of more than ninety percent of MSN, which is densely-studded with dendritic spines. Biological experiments studies have showed that the dopamine axon synapses innervating the striatum located on the neck of dendritic spines (Wickens and Arbuthnott, 2005) and glutamatergic synapses from the cortex located in the dendritic spine head. Dopamine receptors include D1 receptors and D2 receptors, which all distribute on dendritic spines. We considered dopamine produced by the substantia nigra to stimulate D1 and D2 receptors in the striatum, and its effect was not simply excitatory or inhibitory, but a modulatory (Frank et al., 2007). To simplify the computational model, it was reasonable to hypothesize that dopamine excited the direct pathway through the D1 receptor and inhibited indirect pathway activity by D2 receptors. Experimental studies have found that the main dendrites are not covered by spines, while the secondary dendrites and subordinate dendrites are inlaid with spines in varying densities (Graveland et al., 1985; McNeill et al., 1988). Therefore, we assumed that there were 32 glutamate synapses and 32 dopamine synapses on D1 and D2, respectively, which were located at the subordinate dendrites. Since the shape of dendritic spines is very small, the influence of the topology of the dendritic spines on the entire network was ignored, only the changes about the signals from the dopaminergic neurons and the cerebral cortex were taken into account. Therefore, we applied the method of removing synapses from dendritic spines to simulate the shedding of dendritic spines. The number of spine loss is denoted by Ns, then percentage of dendritic spine loss is expressed as Ns/32. For the case of dendritic trees, the degradation of dendritic trees was simulated by disconnection of adjacent two dendrites. At this point, we not only need to consider the ability of MSN to receive signals, but also the impact of changes in the MSN topology on the network. There were two factors that affect the input of the basal ganglia on thalamic—the total proportion of the removed dendritic spines and the total proportion of the degraded dendrites trees. For the sake of simplicity, we used the number of removed dendrites, denoted by Nd, were divided by all secondary and subordinate dendrites (there are 32) to express the percentage of dendrites tree degeneration (Nd/32).

**Figure 2:**
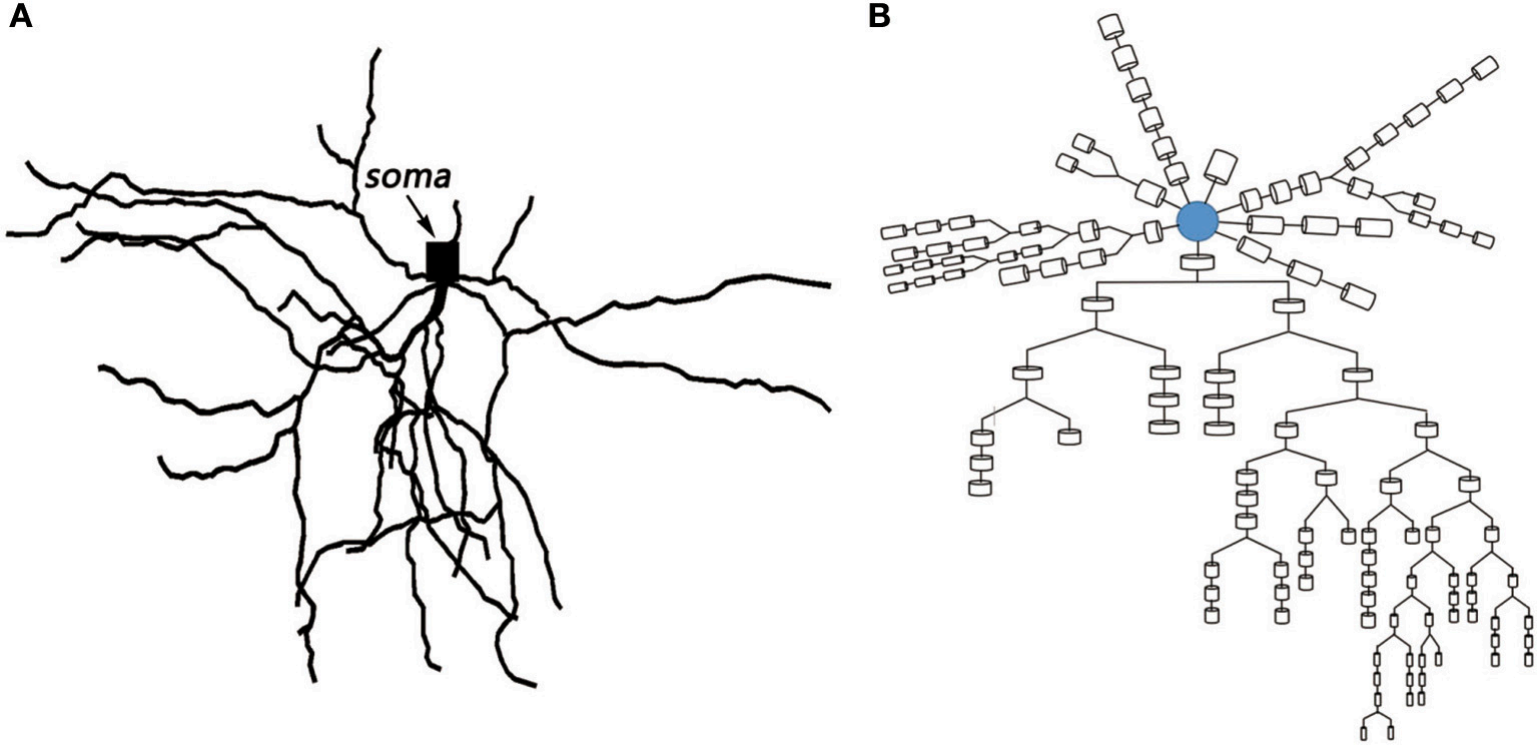
**(A)** Topological structure of medium spiny neuron. **(B)** Corresponding multi-compartment model.

Based on the anatomical experiments of animals and the electrophysiological parameters of different compartments, the MSN model constructed in this paper was very similar to the neuronal cells of the actual mice. The relevant biological experimental data were derived from published studies (Martone et al., 2003; Ascoli et al., 2007). The spatial topology and the corresponding multi-compartment model are shown in the following Figure 2A,B. The model with 121 compartments includes a cell body and 58 dendrites. The model of soma incorporates intrinsic 14 ion channels and leakage current known to be expressed in MSNs. There are two types of sodium channels: fast *I*_*F*_ and persistent sodium *I*_*P*_, six types of potassium channels: fast *I*_*Af*_ and slow A-type potassium *I*_*s*_, 4-AP resistant persistent potassium *I*_*KRP*_, inwardly rectifying *I*_*KIR*_, as well as small *I*_*SK*_ and large *I*_*BK*_ conductance calcium-dependent potassium current, six types of calcium channels: N- types, Q- types, R- types, and T- types calcium currents, high voltage activated *I*_*L*12_ and low voltage activated L-type *I*_*L*13_ calcium currents. The dendrites contain all the ion channels in the cell body except *I*_*KRP*_, which is derived from the references (Steephen and Manchanda, 2009). The biophysical parameters of the model were derived from data that has been published (Wolf et al., 2005).

In the theoretical analysis, we adopted conductance-based Hodgkin–Huxley models to describe the electric properties of the single cell. The internal electrical signal transmission of neurons was based on Rall's cable model. We used a discrete format of Rall's cable model to characterize the connection between different compartments. The current balance equation of each compartment model was described as:

(7) $$C_{m}\frac{dV_{i}}{dt}=-I_{ion}+I_{ext}+\frac{V_{i+1}-V_{i}}{R_{i+1,i}}-\frac{V_{i}-V_{i-1}}{R_{i,i-1}}$$

Here, *V*_*i*_ represents the membrane potential of the *i*th compartment and *I*_*ion*_ is ionic currents of the *i*th compartment. *R*_*i*+1, *i*_ and *R*_*i,i*−1_ represents connection strength of compartment interchange currents connected. *I*_*ext*_ represents the current induced by external stimuli. According to the multi-compartment model described above, membrane potential (υ_*msn*_) of the soma was determined by the current balance equation:

(8) $$\begin{array}{l} C_{m}\upsilon_{msn}\prime =-I_{NaF}-I_{NaP}-I_{KAf}-I_{KAs}-I_{KIR}-I_{KRP}-I_{BK}\\ \;-I_{SK}-I_{CaL12}-I_{CaL13}-I_{CaN}-I_{CaQ}\\ \;-I_{CaR}-I_{CaT}-I(t)-I_{cor\to msn}-I_{DA\to msn} \end{array}$$

There are eight compartments connected to the soma. *I*(*t*) represents these compartment interchange currents connected to the soma. The current *I*_*cor*→*msn*_ denotes the excitatory synaptic input from the cerebral cortex. For D1, *I*_*DA*→*msn*_ represents the excitatory input from dopaminergic neurons, for D2, which represents the inhibitory input from dopaminergic neurons.

### 2.3. Synaptic current

AMPA, NMDA, and GABA currents were modeled using a modified two-state synapse which based on the Exp2Syn synapse in NEURON, These currents obeyed the following equation:

(9) $$I_{syn}={\bar{g}}_{syn}(h-m)(V-E_{syn})$$

(10) $$m^{\prime }=\frac{m}{\tau_{on}},h^{\prime }=\frac{h}{\tau_{off}}$$

Where τ_*on*_ is the rise time and τ_*off*_ is the decay time of the synapse, Decay and rise time constants for NMDA and AMPA channels were taken from the published reports (Gotz et al., 1997), and GABA time constants were taken from neocortical pyramidal cell data (Galarreta and Hestrin, 1997). *E*_*syn*_ is the reversal potential, ḡ_*syn*_ is the maximal synaptic conductance. NMDA and AMPA reversal potentials were set to accepted values of 0*mV* (Moghaddam, 2003), whereas reversal potentials for GABA was set to −80 mV to match published reports (Tepper et al., 2004). The specific parameters value are described in Table 3 in Supplementary Material. The synaptic current from α to β neuron therefore was calculated using:

(11) $$I_{\alpha \to \beta }=g_{\alpha \to \beta }\times I_{syn}$$

Here, *g*_α → β_ is the synaptic connection weight. The specific parameters are described Table 4 in Supplementary Material.

## 3. Network validation

### 3.1. Position selection for dendritic spines loss

**Figure 3:**
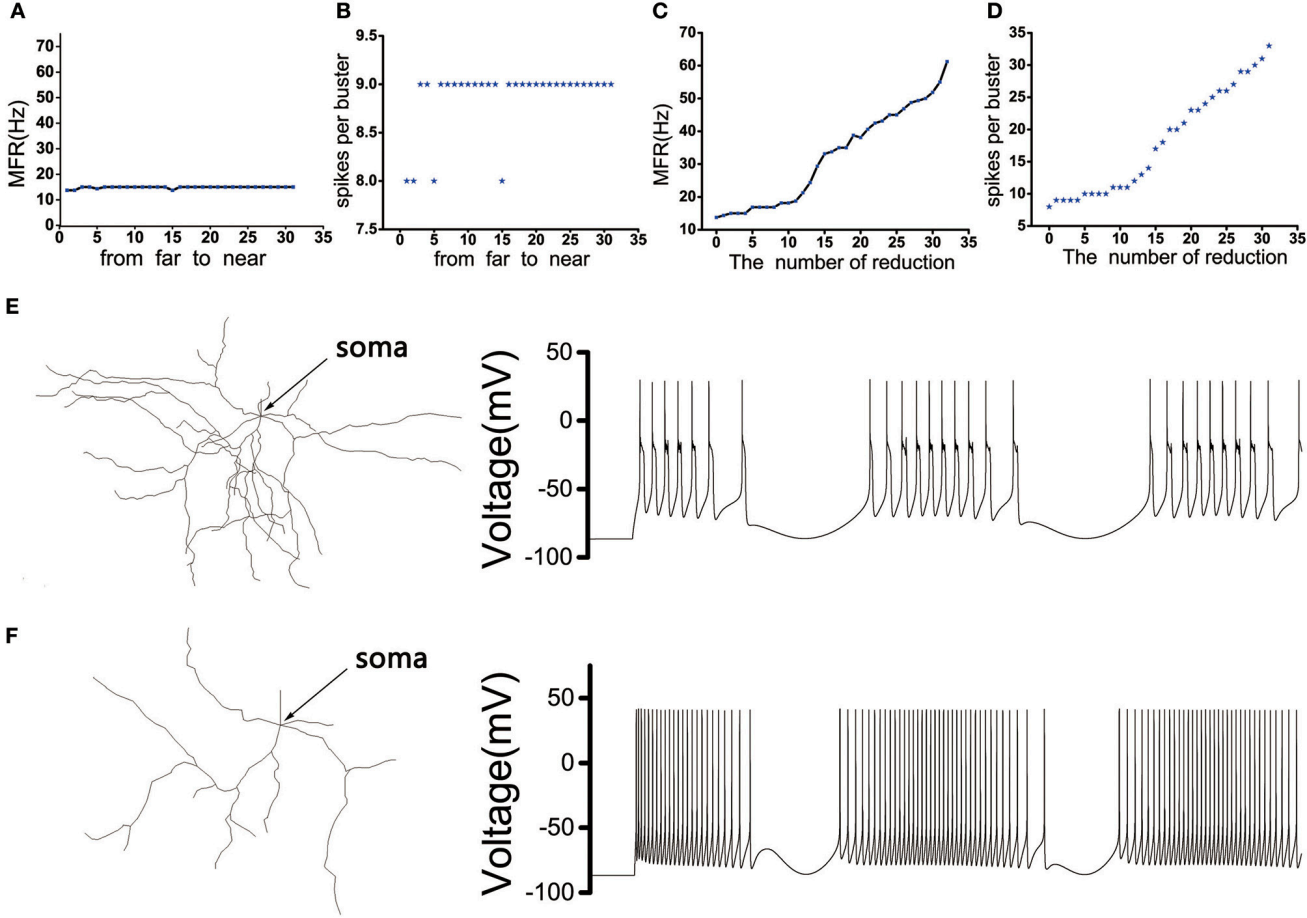
**(A)** The influence of the distance between the removed dendrites and the soma on the MFR of MSN. **(B)** The influence of the distance between the removed dendrites and the soma on spikes per burst. **(C)** The effect of the number of dendrites degradation on discharge of MSN. **(D)** The effect of the number of dendrites degradation on spikes per burst. **(E)** The figure on the left is the topological structure of MSN and the right is corresponding discharge pattern when the number of removed dendrites is 5. **(F)** The topological structure of MSN and corresponding discharge pattern when the number of removed dendrites is 32.

The position of the dendritic spines loss we chose was random. In order to eliminate the effect of the position, we need to discuss the influence of the position where the dendrites were removed on the discharge of the MSN cell body. The model showed that the discharge activity of the cell body remained essentially unchanged under the same stimulus, whether the distal dendrites or the proximal dendrites (closer to the soma) were removed (Figures 3A,B). In addition, as the number of degraded dendrites increased, the mean firing frequency (MFR) of MSN increased significantly (Figures 3C,E,F), as well as the number of peaks in each burst (Figure 3D).

### 3.2. Model neuron firing rate

**Figure 4:**
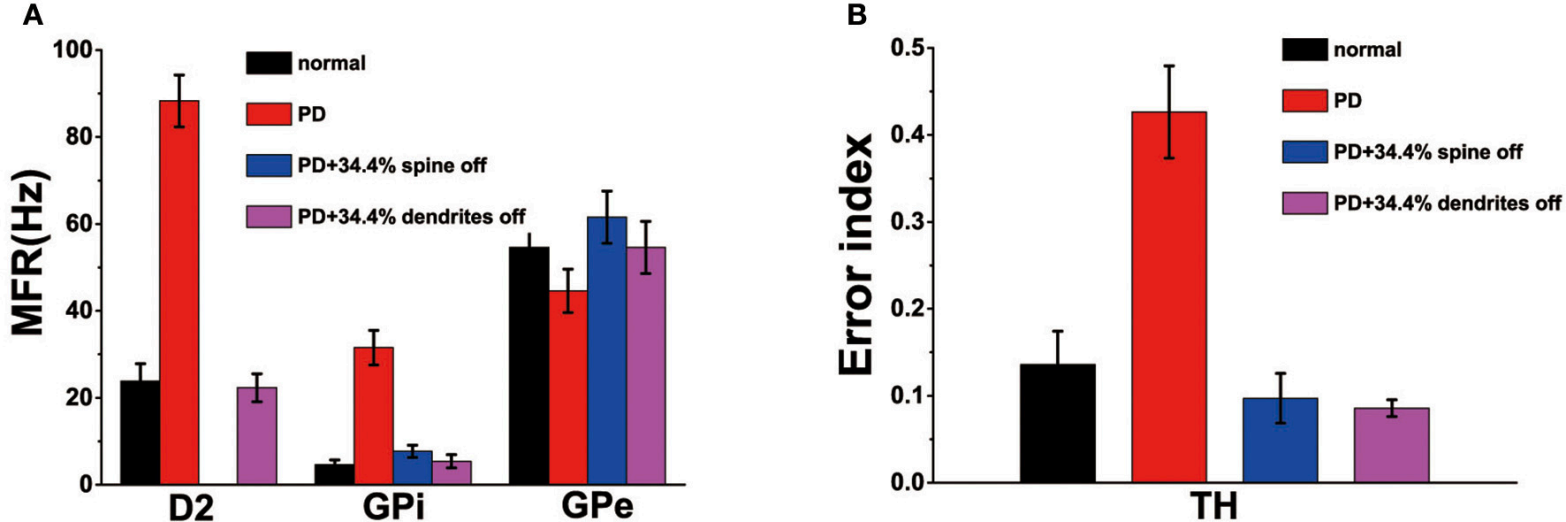
**(A)** The mean firing rates for D2, globus pallidus interna (GPi) and externa (GPe) cells under normal, Parkinson's disease (Dopaminergic neurons is reduced by 50%), PD with 34.4% spine loss, and PD with 34.4% dendrites degradation conditions. **(B)** The error index for TH under normal, Parkinson's disease, PD with 34.4% spine loss and PD with 34.4% dendrites degradation conditions. Standard deviation bars for model data are shown for ten 16-s simulations under each condition.

Recordings in monkeys treated with 1-methyl-4-phenyl-1,2,3,6-18 tetrahydropyridine (MPTP), similar to the emergence of Parkinson's disease, indicated that the frequency of spontaneous discharges increased in the striatum neurons (Bamford et al., 2004). Our model shown that the loss of spines increased the MSN firing rate increases (Figure 3C). Therefore, compared to normal conditions, the model of MSN showed a higher spontaneous firing rate in the PD state. In addition, published experimental results have demonstrated that the firing rate increased in the GPi, decreased in the GPe after administration of MPTP (Boraud et al., 1998; Wichmann and Soares, 2006). Similarly, the firing rate of D2 in PD state was higher than in the normal state(Pang et al., 2001; Mallet et al., 2006). In the network model constructed in this paper, the changes in mean firing frequency of these neurons were consistent with the previous experimental results (Figure 4A).

### 3.3. Effect of dopamine depletion on MFR of TH

**Figure 5:**
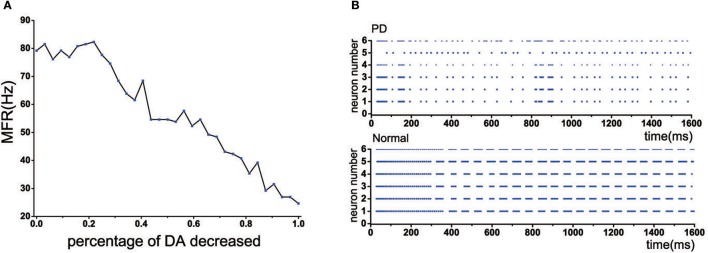
**(A)** The mean firing rate of TH with dopamine reduction. **(B)** Discharge patterns of TH neurons. Rastergrams plots under normal and PD state.

Experimental studies have revealed that GPi, the output nuclei of the basal ganglia, became exceptionally active in PD state (Filion and Tremblay, 1991), resulting in increasing the level of inhibition onto the thalamus. In our model, when the dopamine depletion did not exceed 22%, the mean firing frequency of TH was maintained at a normal level. However, when the amount of dopamine decreased by more than 22%, the mean firing frequency of TH was significantly reduced (Figure 5A). In addition, the discharge pattern of TH has also changed, which exhibited an irregular discharge compared to the previous bursting-like firing patterns (Figure 5B).

### 3.4. The effect of spine loss and dendritic trees degradation on TH discharge

**Figure 6:**
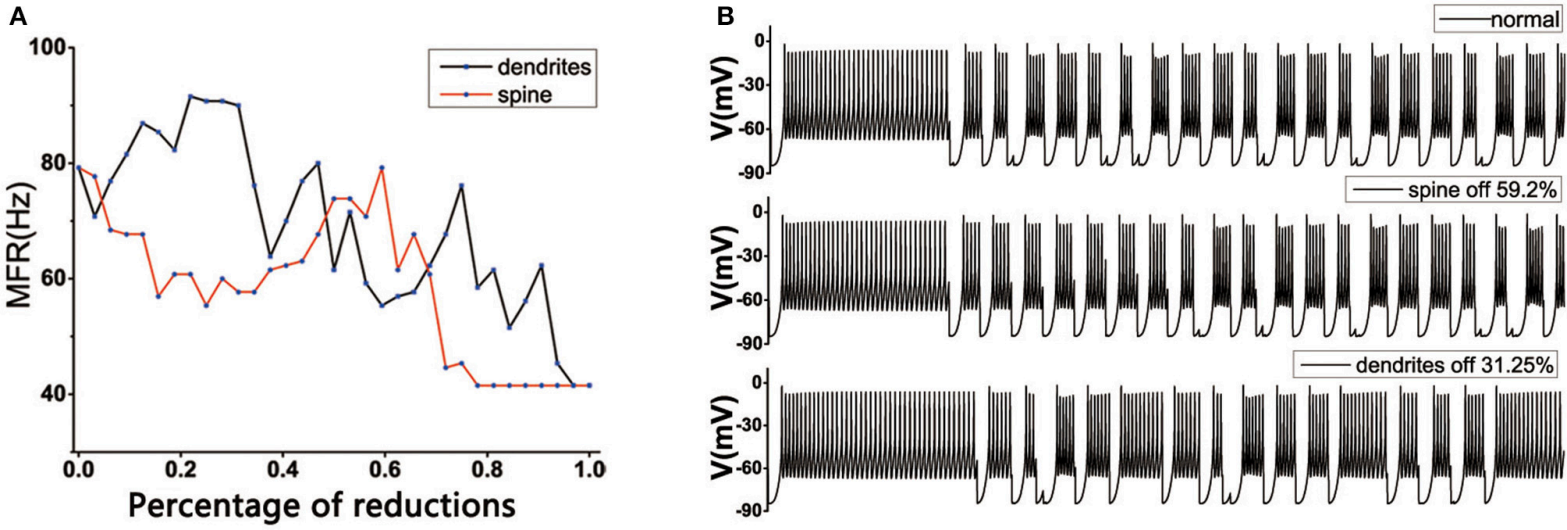
**(A)** Effect of dendritic spine and dendrites degeneration on mean firing rate of the thalamic cells in the BG model under the normal state. With the increase in the number of dendritic spines loss and dendrites degeneration, the average discharge rate is subject to complex changes. The horizontal axis represents the percentage of degraded dendrites and spines. **(B)** The discharge diagrams of TH in different states. The figure above shows the normal structure (MSN has a complete topology). The middle of the figure shows the situation of spines loss to 59.2%. The figure below shows the state of dendrites shedding to 31.25%.

In the normal state, the MSN's topology was complete in striatum, the loss of spines would change the mean firing rate of TH. TH reached the normal level in both positions: one was the original state, which was very obvious, the other was the dendritic spine loss in the range 50~60% (Figure 6A). However, for the dendritic trees degradation, the situation tended to become more complex. One of the important reasons might be that the dendritic trees degradation not only caused the spines located on the corresponding dendrites to be lost, resulting in a change in the receiving signal of the striatum. At the same time, the degradation of the dendrites itself also caused an increase in the discharge of in the medium spine neurons, and the greater the dendritic degradation, the greater mean firing rate of the cell (Figure 3C). At the beginning, with the dendrites degradation, the discharge of TH increased, the mean firing rate of TH reached the maximum when the dendrites degraded about 15–28%, subsequently fluctuated downward trend (Figure 6A). During this period, the firing rate of TH could return to normal at some points, such as 31.25% (Figure 6B) or 45%.

### 3.5. Error index

In the PD state, due to the reduction of dopamine, the regulation of the substantia nigra on the basal ganglia was impaired, the electrical signals transmitted to the effector were lost or incomplete. Therefore, the EI indicator of the PD patients was greater than the normal people (Figure 4B), which might be attributed to a decrease in the number of normal peaks or an increase in the number of incomplete peaks.

## 4. Result

Now, we used the validated model to detect the effect of the proportion of spines loss and dendrites degradation on the whole network.

### 4.1. Electrical activity in cortical neuron under electromagnetic induction

As one of the primary information input nuclei to the basal ganglia, the discharge characteristics of the cortical neurons are crucial for BG network. We considered the cortical neuron model with electromagnetic induction, and magnetic flux φ was imposed on the cortical neuron to describe the effect of electromagnetic induction. Moreover, the effects of alternating current electric field induced by magnetic stimulation were also considered to explore the mode transition of electrical activities. The improved neuron model is described by the following dynamical equation:

(12) $$\begin{array}{l} \left\{ \begin{array}{l} C_{m}\upsilon_{cor}\prime =-I_{leak}-I_{Na}-I_{Kd}-I_{M}-I_{L}-A_{e}\omega C_{m}cos(\omega t)\\ \;+I_{app}+k\rho (\varphi )(\upsilon_{cor}+V_{e}),\\ \;y^{\prime }=\alpha_{y}(\upsilon_{cor})(1-y)-\beta_{y}(\upsilon_{cor})y,\;(y=m,h,n,q,r,p) \end{array}\right.\\ \;\varphi^{\prime }=k_{1}\upsilon_{cor}-k_{2}\varphi , \end{array}$$

(13) $$\left\{ \begin{array}{l} \rho (\varphi )=(\alpha +3\beta \varphi^{2}),V_{e}=A_{e}sin(\omega t)\\ I_{leak}=g_{l}(\upsilon_{cor}+V_{e}-E_{l}),\\ I_{Na}=g_{Na}m^{3}h(\upsilon_{cor}+V_{e}-E_{Na}),\\ I_{Kd}=g_{Kd}n^{4}(\upsilon_{cor}+V_{e}-E_{Kd}),\\ I_{L}=g_{L}q^{2}r(\upsilon_{cor}+V_{e}-E_{L}),\\ I_{M}=g_{M}p(\upsilon_{cor}+V_{e}-E_{M}), \end{array}\right.$$

Where φ, *V*_*e*_ represents the magnetic flux across the membrane and additive induction membrane induced by external magnetic stimulation, respectively. In fact, *V*_*e*_ = *A*_*e*_*sin*(ω*t*) is alternating current electric field mentioned above, which can be seen as an additive perturbation to the membrane potential (*V*_*cor*_) (Yu et al., 2013). This time, we change υ_*cor*_ to υ_*cor*_+*V*_*e*_, and $C_{m}\frac{dV_{e}}{dt}={\left(Aesin\left(\omega t\right)\right)}^{\prime }=A_{e}\omega C_{m}cos\left(\omega t\right)$. This is what the right side of the Equation (12) describes. The function ρ(φ) is the memory conductance used to describe the relationship between magnetic field and membrane potential of neuron, which develop from the magnetic flux-controlled memristor (Bao et al., 2010). *I*_*app*_ represents the external forcing current, and here we only consider the direct-current type. *k*, *k*_1_, *k*_2_ are parameters that describe the interaction between membrane potential and magnetic flux. α and β are two positive constant parameters. Detailed description of these parameters can refer to the literature (Lv et al., 2016), and the other parameters such as ω, *A*_*e*_, and *I*_*app*_ are differently changes. The membrane capacitance and the gate variable for channels will remain the same.

**Figure 7:**
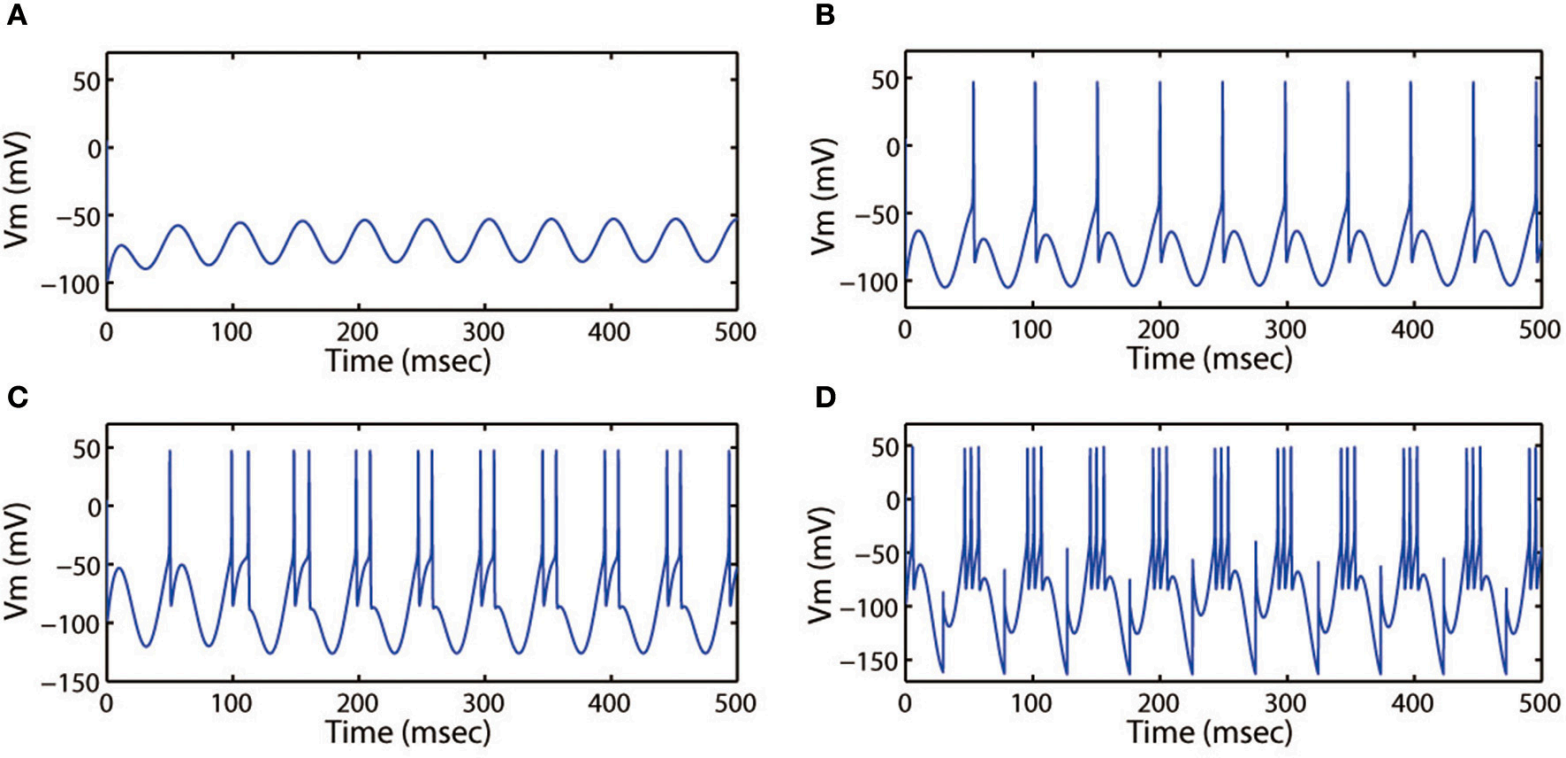
Action potential of neuron in neuron under different amplitude of induced electric field. **(A)** *A*_*e*_ = 0.4, **(B)** *A*_*e*_ = 0.8, **(C)** *A*_*e*_ = 1.2, **(D)** *A*_*e*_ = 2.0. The external forcing current and frequency are selected as *I*_*app*_ = 0, ω = 6.31, respectively.

First, we discussed the modes of electrical activities induced by the alternating current electric field and without external forcing current being considered. We selected *k* = 0.001, *k*_1_ = 0.1, *k*_2_ = 1, α = 0.4, β = 0.01, and fixed ω = 6.27Hz. Then multiple discharge modes in cortical neuron could be observed through tuning the intensity (*A*_*e*_) of AC electric field, and the time series of membrane potential with different values of *A*_*e*_ were plotted. In Figure 7, when a small intensity *A*_*e*_ = 0.4 acted on the membrane, the discharge patterns of the cortical neuron are subthreshold membrane potential oscillation (SMPO). As the intensity increased, the SMPO state of the cortical neuron was excited into a single spiking state. Further, when the intensity *A*_*e*_ was increased to 2.0, the spiking state with one spike of the discharge patterns is transited to have more spikes.

**Figure 8:**
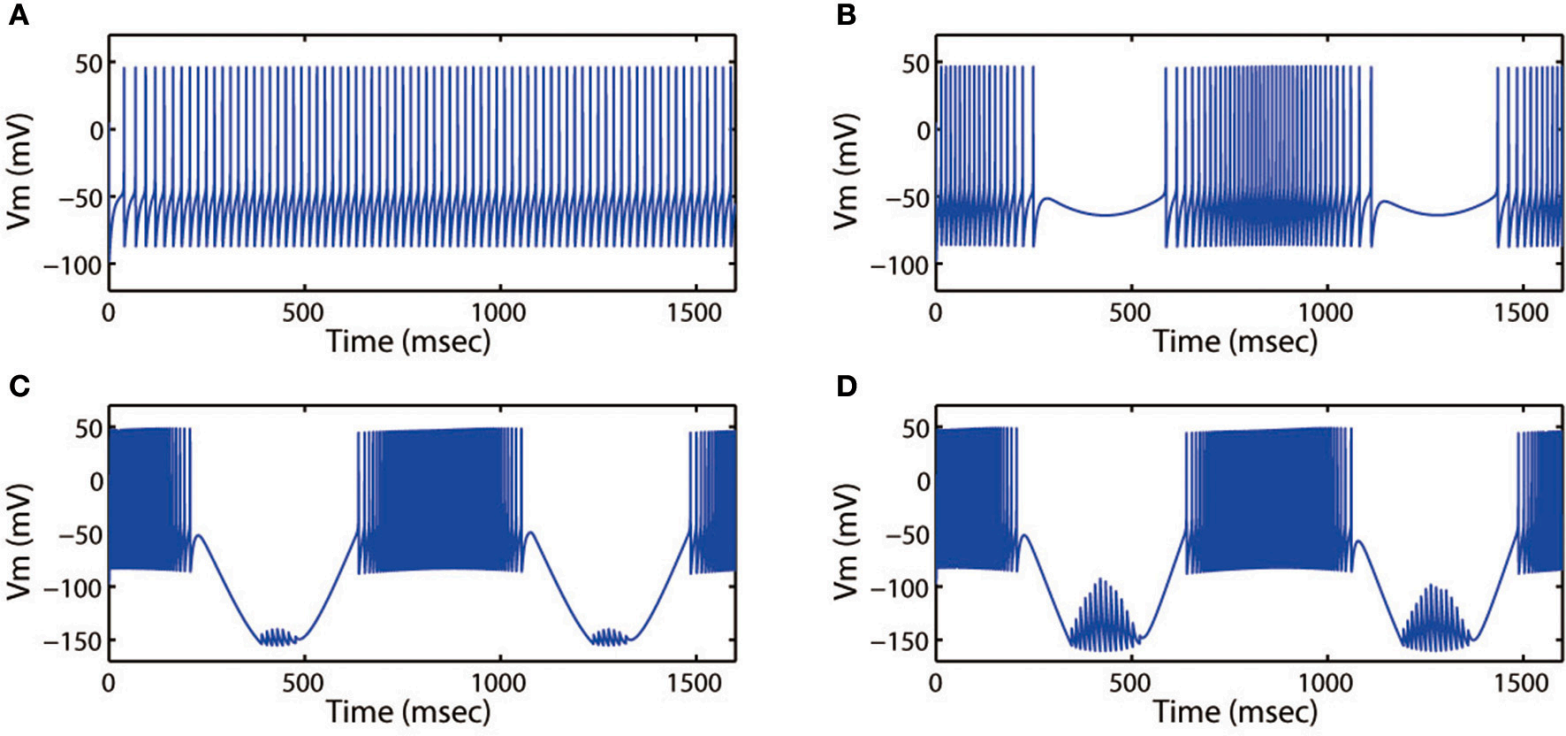
Action potential of neuron in neuron under different amplitude of induced electric field. **(A)** *A*_*e*_ = 0, **(B)** *A*_*e*_ = 0.4, **(C)** *A*_*e*_ = 1.4, **(D)** *A*_*e*_ = 1.6. The external forcing current and frequency are selected as *I*_*app*_ = 3.0, ω = 5.2, respectively.

Next, we discussed the modes of electrical activities when external forcing current was considered, here *I*_*app*_ = 3.0. Fixing ω = 5.2 Hz, variations in stimulus intensity *A*_*e*_ could also generate multiple fring patterns, the results for neuronal action potentials were shown in Figure 8. With the increase of *A*_*e*_ from 0 to 1.6, the discharge patterns transited from period-1 fring to bursting, then turned into a mixed-mode oscillations. Moreover, Figures 7, 8 illustrated that bursting was more prefer to occur when *A*_*e*_ was large, and a small value of *A*_*e*_ would cause the cortical neurons to become spiking or quiescent or subthreshold membrane potential oscillation.

**Figure 9:**
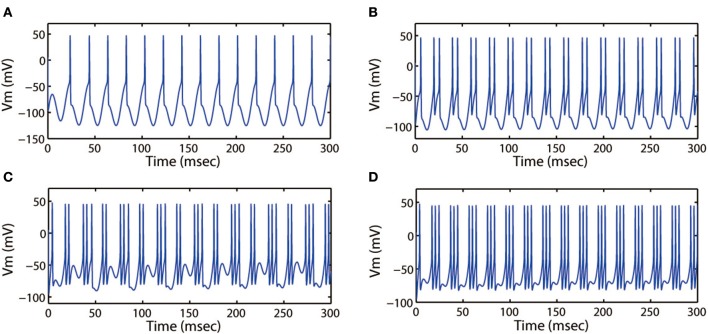
Action potential of neuron in neuron under different values of external forcing current *I*_*app*_. **(A)** *I*_*app*_ = 0, **(B)** *I*_*app*_ = 5.0, **(C)** *I*_*app*_ = 9.0, **(D)** *I*_*app*_ = 11.0. The amplitude and frequency of induced electric field are selected as *A*_*e*_ = 1.2, ω = 6.28, respectively.

Then we explored other interesting modes of the discharge patterns. For *k* = 0.001, *k*_1_ = 0.1, *k*_2_ = 1, α = 0.4, β = 0.01, *A*_*e*_ = 1.2, ω = 6.28, Figure 9 showed that the firing patterns in cortical neuron were much dependent on the external forcing current. As the external forcing current was increased from 0 to 11.0 displayed in Figures 9A–D, we could found that the electrical activities transited from spiking to period-2 bursting, then from chaotic bursting to period-3 bursting. That is to say, the number of spikes in each burst increased with increase of stimulus intensity.

### 4.2. Effect of dendritic spines loss on EI

Studies have shown that the clinical symptoms of Parkinson's disease occured when the death of dopaminergic neurons in substantia nigra was more than 50% and the loss of dopamine in the striatum exceeded 80%. In response to the reduction of dopamine, some excitatory input synapses as well as their postsynaptic spines were lost (Stephens et al., 2005). Day et al. also found that selective loss of glutamatergic synapses would appear in MSN when the concentration of dopamine in the striatum was reduced (Day et al., 2006). Therefore, it was a reasonable assumption that dendritic spines were preferred to fall off at the location where the binding site of dopamine receptor had been lost. We divided the dendritic spines from two types: Type-1 indicated that the loss of dendritic spines occured at the site where the dopamine receptor binding site had been lost. Type-2 indicated that the loss of dendritic spines occured at sites where the dopamine receptor binding site has not been lost. According to our assumptions, we could divide the dynamic changes of the BG network into two stages: First, the dendritic spines lost in the type-1 position, and the corresponding glutamatergic synapses were also lost, protecting D2 against too much excitation, reducing the inhibition of indirect pathway on TH. Second, type-2 spines began to fall off, the excitatory input from the cortex to D2 was decreased, while the inhibitory input from dopaminergic neurons was also reduced. The discharge activity of D2 would not always be enhanced or weakened, but in a fluctuating change. Therefore, the process of dendritic spine loss in fact was a complex regulatory process of basal ganglia.

**Figure 10:**
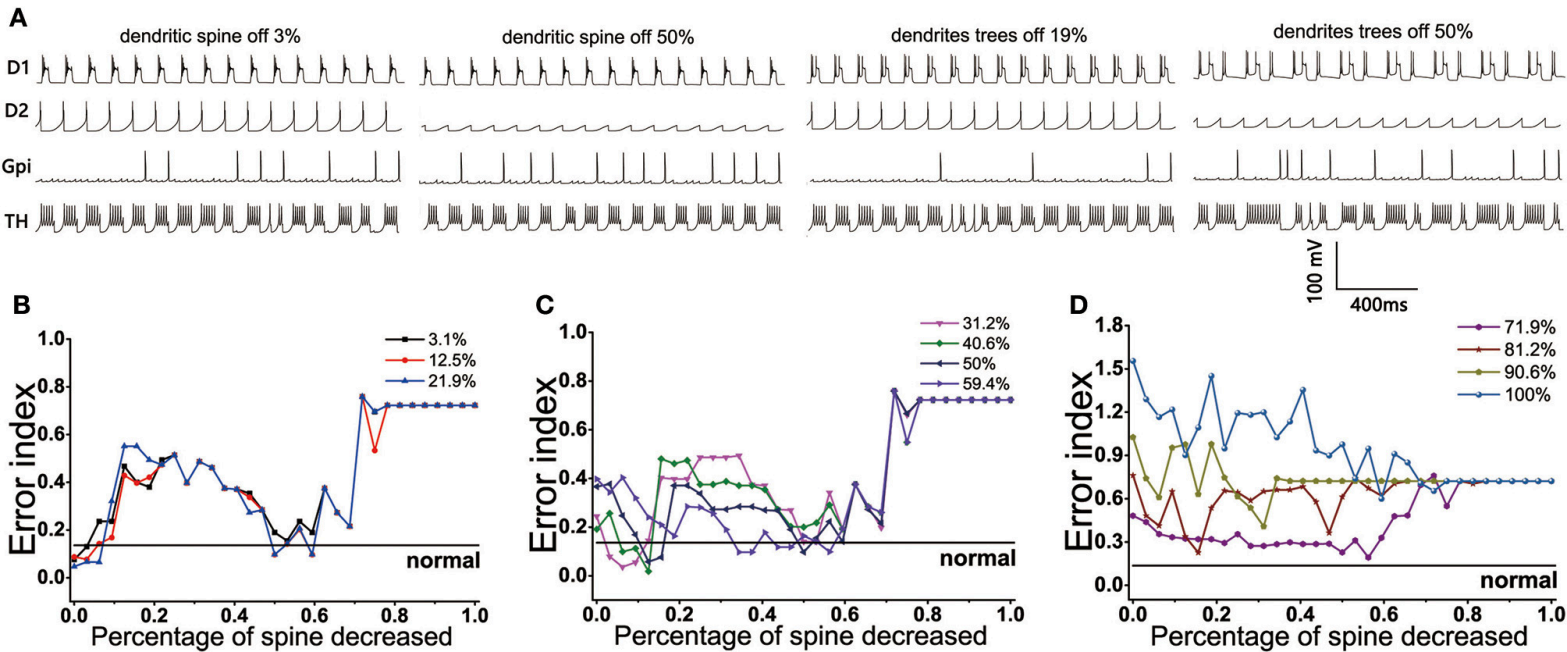
(Top) The firing patterns of D1, D2, GPi, and TH in the case where the dopamine content is reduced by 12.5%. **(A)** Dendritic spines are lost by 3, 50% and dendrites trees are degenerated by 19, 50%. (Bottom) Results from BG model using proportions of dendritic spines loss impact on EI. **(B)** EI changes with the dendritic spines loss when dopamine concentration in the striatum decreased by 3.1, 12.5, 21.9%. EI level can reach the normal value twice: dendritic spines fall off in 0~10% and 50~62%, respectively. **(C)** Dopamine concentration in the striatum decreased by 31.2, 40.6, 50, 59.4%. EI level also can reach the normal value twice: dendritic spines fall off in 9~18% and 50~62%, respectively. **(D)** Dopamine concentration in the striatum decreased by 71.9, 81.2, 90.6, 100%. EI is also reduced, but it cannot be restored to normal levels.

The BG network model we constructed showed that the loss of spines could bring the EI index to the normal level under the circumstance of the dopamine concentration decreasing by no more than 60%. The reduction of dopamine was different, the changes of EI index with the dendritic spine loss were also different. Here, the cases could be divided into three types: First, when the amount of dopamine depletion did not exceed 22% (Figure 10B), the EI index of the model did not increase significantly, suggesting that it could not cause disorder about the regulatory functions of basal ganglia. However, a small amount of spines loss made the EI index rise, one of the possible reasons was the reduction of D1 activity with the loss of glutamatergic synaptic contacts, making the function of the direct path transmitting signal weakened. With the dendritic spine further loss (approximately in the range of 47~62%), glutamate excitatory utility and dopamine inhibition achieved a dynamic balance, thus the regulatory function of the basal ganglia temporarily returned to normal. The function of the basal ganglia again disorder when the amount of dendritic spine loss more than 70%. At this time, the connection point of the dopamine receptor was greatly reduced, and its effect was equal to the reduction in dopamine concentration. Second, the EI index of the BG network reached the normal level in two ranges when the concentration of dopamine was decreased by 30~60% (Figure 10C). One place that made the EI reach the normal level was the spines reduced by 9~18%. A reasonable explanation was that the amount of dopamine reductions was relatively higher, the type-1 spines were first lost, and the activity of D2 was reduced because of a reduction about excitatory input to D2, the important regulatory function of the indirect pathway was restored so that cerebral cortical information was regulated by the basal ganglia and communicated correctly to the effector (TH). At this moment, a phenomenon that the lower the degree of dopamine damage, the less spines that need to lose to return to normal EI could also be observed. Another range that made the EI reach the normal level was the spines reduced by 47~62%. At this time, type-1 dendritic spines have been completely lost and type-2 spines began to fall off, thus the proper ratio of excitatory stimulus and inhibitory stimulus was established for optimal basal ganglia regulatory function. Third, the dendritic spines loss have already not been able to make EI reach the normal level when the amount of dopamine decreased up to 70% (Figure 10D).

### 4.3. Effect of dendrites tree degeneration on EI

**Figure 11:**
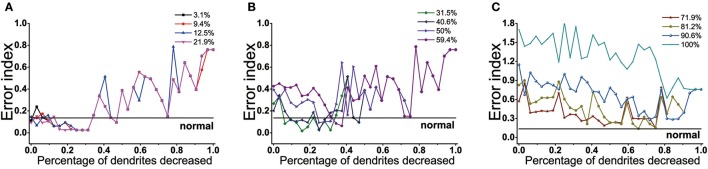
Results from BG model using proportions of dendrites trees degeneration impact on EI. **(A)** EI changes with the dendrites trees degeneration when dopamine content in the striatum decrease by 3.1, 9.4, 12.5, 21.9%. When dendrites trees are reduced by 0–34%, EI is maintained at normal level. **(B)** Dopamine content in the striatum decrease by 31.2, 40.6, 50, 59.4%. In the dendrites shedding range of 9~34%, EI is maintained at normal level when the dopamine content is reduced by 30 and 40.6%. the dendrites degeneration range of 34~40% corresponds to 50% of the dendritic degradation, and 45~50% corresponds to 59.4%. **(C)** Dopamine content in the striatum decreased by 31.2, 40.6, 50, 59.4%. In the dendrites shedding range of 9~34%, EI is maintained at normal level when the dopamine content is reduced by 30 and 40.6%. the dendrites shedding range of 34~40% corresponds to 50% of the dendritic degradation, and 45~50% corresponds to 59.4%.

The effect of dendrites tree degeneration on EI was also significant. In the case where the dopamine reduction was not more than 22%, the EI could be maintained at a normal level or even below the normal level as long as the dendrites trees degradation were no higher than 34% (Figure 11A). With the further degradation of the dendrites, the EI gradually rose, which might drop and approach the normal value at some point in the future. But this was only a case, the overall EI was still showing a rising trend.

When the dopamine concentration decreased by 30~60% (Figure 11B), EI was significantly increased, indicating that the basal ganglia appeared dysfunction and could not effectively transmit the signal. This moment, dendrites trees degradation could protect D2 from too much glutamate excitatory stimulation, reducing the activity of D2, thus restoring the regulation of indirect pathway. Different with dendritic spines loss, the elimination of the glutamatergic synaptic contact also coincided with the shrink of dendritic trees in D2, resulting in D2 activity was also affected by its topological structure. In addition, it could be seen that the lower the dopamine content, the more the amount of dendrites trees degradation required for EI to achieve normal values for the first time. In addition to some points would decline, EI overall showed a fluctuations rise trend in the case where the amount of dendrites trees degradation was over 50%. When dopamine decreased up to 70% (Figure 11C), the dendrites trees degradation could not make the function of the basal ganglia back to normal even if it made EI decrease. The results also showed that dopamine concentration was different, the range of dendrites trees degradation which made the EI reach the normal level was not the same. The lower the dopamine concentration, the shorter the length of interval maintaining EI at customary levels.

### 4.4. Effect of cortical input under dopamine depletion

**Figure 12:**
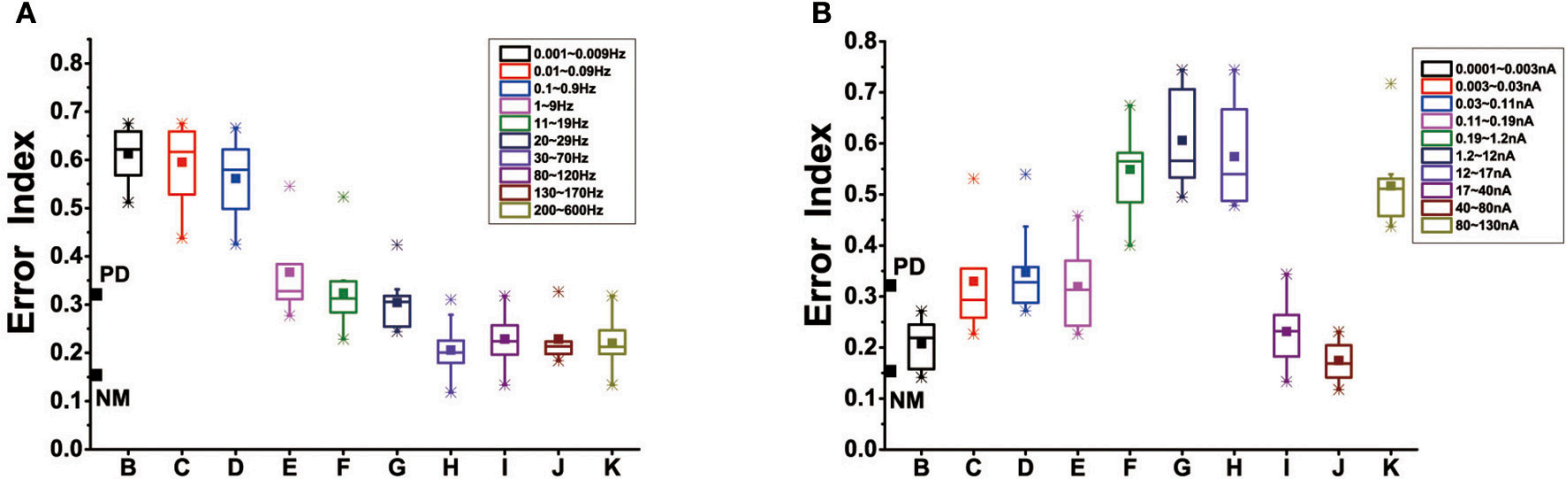
**(A)** Effect of stimulus frequency on EI of the thalamic neurons in the BG model, based on 10 trials which are selected randomly of 1,600 ms in each box. Here, we divide the range of 0.001~600 Hz into 10 representative sections, corresponding to ten different frequency ranges. **(B)** Effect of stimulus amplitude on EI of the thalamic neurons, we divide the range of 0.0001~600 nA into 10 representative sections, corresponding to ten different amplitude ranges. The boxes show the minimum, first quartile, median, third quartile, and maximum for the 10 trials in each case.

Modulation of cortical activity might prevent dopamine depletion-induced dendritic spines loss. Box-plots were obtained from ten trials of 16 s each. The stimulus form that we chose was alternating current electric field, given by the equation: *I*_*stim*_=*Asin*(ω*t*), where *A* corresponds to stimulation amplitude, ω to stimulation frequency. We attempted to explore the effect of different levels of stimulus frequency, amplitude in the case of dopamine reduction of 50%. Figure 12 showed that in our results, stimulus frequency <30 Hz did not attain improvement in basal ganglia dysfunction evoked by dopamine depletion. Further, stimulation at quite low frequencies (about 0.001~0.9 Hz) actually led to further disorders. As the stimulus frequency increases, approximately 1–30 Hz, the error index remained at the same level as the PD state. Unlike the effects of stimulus frequency with a almost monotonous dependence, the effect of stimulus amplitude on the network was irregular. When the intensity of the stimulus was <0.003 nA, that was very low, EI could restore to the healthy level. Moreover, EI significantly also dropped for the stimulus amplitude between 17 and 30 nA, and saturated at 80 Hz. Stimulus intensity between 0.003 and 17 nA or strong enough (>80 nA) in cortical neuron resulted in little or no improvement of basal ganglia dysfunction.

## 5. Discussion and analysis

### 5.1. Effects of the spine loss and dendrites trees degradation on downstream neurons

The model we constructed showed that the basal ganglia could be restored to normal function in some (more than one) ranges of dendritic spine loss under the same dopamine depletion. When the amount of dopamine decreased in the range of 3~21.9%, there were two ranges of the dendritic spine loss, 0~10% and 50~62%, respectively, making the EI indicator reduce to normal level. However, their impact on the downstream neurons were not the same. The discharge patterns of GPi, D1, D2, TH were observed when the concentration of dopamine was reduced by 12.5% (Figure 10A). It could be seen from the discharge diagram of GPi that the loss of dendritic spines could effectively inhibit GPi activity, thereby reducing its inhibition on TH.

D1 activity was higher in the case of dendritic spines reduced by 30%, compared to reducing by 50%, indicating that the excitability of the direct pathway to thalamus of the former was greater than the latter. At the same time, D2 activity was also higher in the case of dendritic spines reduced by 30%, showing that the inhibition of the indirect pathway to the thalamus of the former was also stronger than the latter. The combined effects of these two factors eventually led to a fact that their effects of basal ganglia on thalamus were almost equivalent, although the principles were distinct. Similarly, when the amount of dopamine decreased by 31.2~59.4%, the loss of dendritic spines could restore the basal ganglia to normal function in two places as well. The principle was similar to the above discussion.

Considering the degradation of dendrites trees (Figure 10A), the EI index was the same in the case of a 19 and 50% degradation when the concentration of dopamine decreased by 12.5%. D1 activity was higher in the case of dendrites degraded by 19%, compared to degrading by 50%. However, the D2 activity of the former was also significantly higher than the latter. The direct pathway of the former had a stronger excitatory effect on thalamus, and the inhibitory effect of the indirect pathway on thalamus was also stronger. Both states could be linked and might well play the decisive role in normal function of the basal ganglia.

### 5.2. Phase diagram

**Figure 13:**
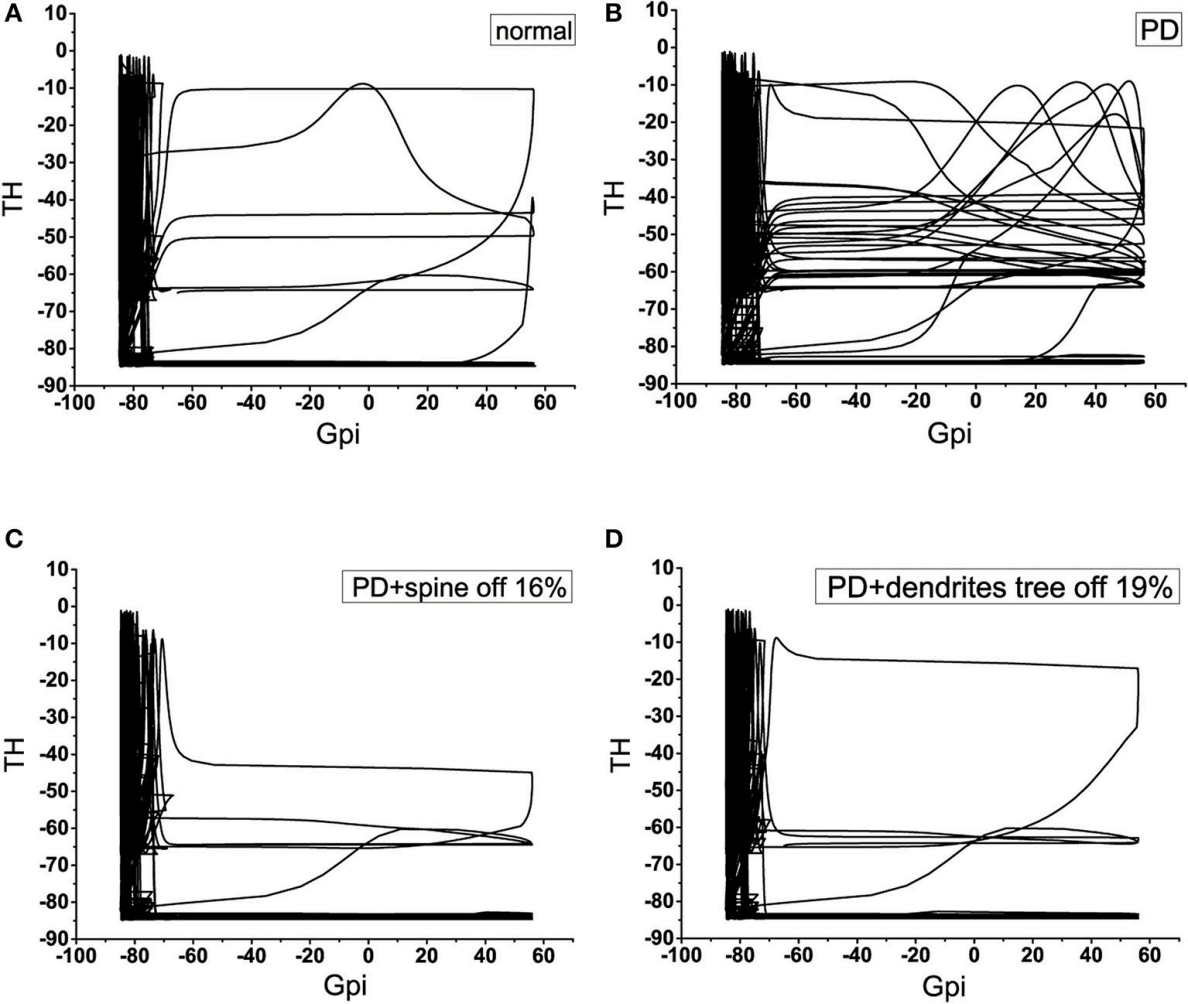
Four classes of phase diagram about the GPi-thalamic neuron channel. The abscissa indicates the action potential of GPi, and the ordinate indicates the action potential of TH. **(A)** Normal state. **(B)** Parkinson's disease state. **(C)** PD with 16% spine loss. **(D)** PD with 16% dendrites tree degradation.

We applied the phase diagram to gain insight into the mechanism of signal degradation. Since GPi is the output nucleus of the basal ganglia, the signal from the cortex is transmitted to the thalamus through the integration of the basal ganglia. Therefore, in this analysis, we detected how thalamic cells responded to inhibition of GPi neuron. According to the phase diagram proposed numerical results presented above section by using GPi-thalamic channel. Here, we considered four types of GPi neuron input: these would be known as the normal, PD, 16% of the dendritic spines loss in PD state, and 19% of the dendrites tree degeneration in PD state. The phase diagram provided a very powerful way to understand how the signal was attenuated during transmission. In the PD state, the amplitude range of TH was larger in the case where the amplitude of GPi exceeds −70, compared to the normal state (Figures 13A,B). More specifically, the values that could be obtained were very scattered, and there was no specific regular pattern. That is to say, it could not accurately determine the value of TH, causing TH to fail to respond correctly to the output of GPi. These results indicated that there was a problem with the channel GPi-thalamic of the signal output of the basal ganglia. However, when the dendritic spines lost or the dendrites trees degraded to a certain proportion (Figures 13C,D), the phase diagram was basically similar to the normal. That is, thalamus could correctly respond to the inhibition of GPi, thereby slowing down the functional disorder of the GPi-thalamic channel caused by the denaturation of dopaminergic neurons.

### 5.3. Role of cortex in MSN morphology changes dopamine depletion-induced

The loss of dendritic spines is to help confront dopamine depletion, but also at a cost. With the dendritic spines loss, the density of spines on which dopamine receptor binding points are normally located decreased. This would render a Parkinson's disease patient less response to L-dopa (Deutch, 2006), are there any other interventions that prevent MSN morphology change? Early reports suggested that prolonged blockade of excitatory transmission in vivo led to an increase in the density of dendritic spines (Rocha and Sur, 1995). Observations made by Neely et al. who found that spine density and the length of stubby spines had a significant increase in decortication cultures (Neely et al., 2007). They also observed that there were no morphology changes of MSN in cultures where the cortex was stripped when the dopamine was depleted. These findings all supported the hypothesis proposed by Deutch et al. that the cortical played an important role in dopamine-depletion induced degeneration in dendritic spines. Our calculation model showed that the appropriate adjustment of the cortical stimulus intensity and stimulus frequency could restore the basal ganglia to normal function. It was thus stated that proper adjustments to cortical activity did stop the changes in dendritic spines induced by dopamine depleted. These results suggested that modifying cortical drived onto MSN could prevent the decreased responsiveness to L-dopa medical treatment induced by the loss of spines in process of PD, thus leading to effective therapeutics for late-stage parkinsonism. Therefore, our study about cortical activity might provide a new idea on novel clinical therapeutic strategies for Parkinson's disease.

## 6. Conclusion

In this paper, the BG model was based on a biologically similar model of the cortical-basal ganglia-thalamic network, and a simple error index was applied to quantify the normality of thalamic throughput. Using this model, we explored the effect of dendritic spine loss and dendrites trees degradation on basal ganglia function in the case of the different dopamine concentrations and also verified that proper adjustments to cortical activity could prevent these changes in dendritic spines induced by dopamine depleted. Moreover, based on the neuron model of cerebral cortex in the network, an improved Hodgkin–Huxley model was considered by imposing additive memristive current. Using this model, we detected the impact of electromagnetic induction and the dynamical properties of electrical activities. It could be helpful to investigate the influence of electromagnetic induction on BG network and further explore Parkinson's treatment strategy.

## Author contributions

XZ: Designed the study, performed the research, analyzed data, and wrote the paper; SL, FZ, JW, XJ: Contributed to refining the ideas, carrying out additional analyses, and finalizing this paper.

### Conflict of interest statement

The authors declare that the research was conducted in the absence of any commercial or financial relationships that could be construed as a potential conflict of interest.
