# Supplementary material for: The Effects of Medium Spiny Neuron Morphologcial Changes on Basal Ganglia Network under External Electric Field: A Computational Modeling Study
Source: Front Comput Neurosci. 2017 Oct 26;11:91. doi: 10.3389/fncom.2017.00091 (PMC5662631; doi:10.3389/fncom.2017.00091)
Supplement: Supplementary file 1 [file DataSheet1.pdf]

1 SUPPLEMENTARY DATA

Here, we describe the equations of all neurons and the corresponding parameters, as well as synaptic connections. The unit of all membrane voltage are mV, The unit of conductances are mS/cm^2, Currents are expessed in uA/cm^2, time constants in msec, and all membrane capacitance have the unit of $1uF/cm^2$.

TH neuron model

$$C_m {v_{th}}' = -I_L - I_{Na} - I_K - I_T - I_{gpi\to th} + I_{appth},$$
$$I_L = g_L(v_{th} - E_L); I_{Na} = g_{Na} m_\infty(v_{th})^3 h(v_{th} - E_{Na}); I_K = g_K[0.75(1-h)](v_{th} - E_K); I_T = g_T p_\infty(v_{th})^2 r(v_{th} - E_T),$$
$$h' = [h_\infty(v_{th}) - h]/\tau_h(v_{th}), r' = [h_\infty(v_{th}) - r]/\tau_r(v_{th}).$$

STN neuron model

$$C_m {v_{stn}}' = -I_L - I_{Na} - I_K - I_T - I_{Ca} - I_{ahp} - I_{gpe\to stn} - I_{cor\to stn},$$
$$I_L = g_L(v_{stn} - E_L); I_{Na} = g_{Na} m_\infty(v_{stn})^3 h(v_{stn} - E_{Na}); I_K = g_K n^4(v_{stn} - E_K); I_{Ca} = g_{Ca} c^4(v_{stn} - E_{Ca});$$
$$I_T = g_T a_\infty(v_{stn})^3 b_\infty(v_{stn})^2; I_{ahp} = g_{ahp}(v_{stn} - E_{ahp})(\frac{CA}{CA+15}),$$
$$h' = 0.75[h_\infty(v_{stn}) - h]/\tau_h(v_{stn}), n' = 0.75[n_\infty(v_{stn}) - n]/\tau_n(v_{stn}), r' = 0.2[r_\infty(v_{stn}) - r]/\tau_r(v_{stn}),$$
$$c' = 0.75[c_\infty(v_{stn}) - c]/\tau_c(v_{stn}), {Ca_i}' = -0.375 \times 10^{-6}(I_T + I_{Ca} + 22.5 \times Ca_i).$$

GP neuron model

$$C_m {v_{gpi}}' = -I_L - I_{Na} - I_K - I_T - I_{Ca} - I_{ahp} - I_{app} - I_{d1\to gpi} - I_{gpe\to gpi} - I_{gpi\to gpi} - I_{stn\to gpi},$$
$$C_m {v_{gpe}}' = -I_L - I_{Na} - I_K - I_T - I_{Ca} - I_{ahp} - I_{app} - I_{d2\to gpe} - I_{gpe\to gpe} - I_{stn\to gpe},$$
$$I_L = g_L(v_{gp} - E_L); I_{Na} = g_{Na} m_\infty(v_{gp})^3 h(v_{gp} - E_{Na}); I_K = g_K n^4(v_{gp} - E_K);$$
$$I_T = g_T a_\infty(v_{gp})^3 r_\infty(v_{gp} - E_T); I_{Ca} = g_{Ca} s_\infty(v_{gp})^3(v_{gp} - E_{Ca}); I_{ahp} = g_{ahp}(v_{gp} - E_{ahp})(\frac{CA}{CA+10}),$$
$$h' = 0.75[n_\infty(v_{gp}) - h]/\tau_h(v_{gp}), n' = 0.2[r_\infty(v_{gp}) - n]/\tau_n(v_{gp}), {Ca_i}' = -0.1 \times 10^{-5}(I_T + I_{Ca} + 15 \times Ca_i).$$

DA neuron model

$$C_m {v_{DA}}' = -I_{Na} - I_A - I_{KDR} - I_{K,SK} - I_{NaP} - I_{Ca,T} - I_{Ca,L} - I_{Ca,N} - I_{Ca,P} - I_L + I_{appDA},$$

$$I_L = I_{L,Na} + I_{L,K} + I_{L,Ca},$$

$$I_{L,Na} = g_{L,Na}(v_{DA} - E_{L,Na}); I_{L,K} = g_{L,K}(v_{DA} - E_K); I_{L,Ca} = g_{L,Ca}(v_{DA} - E_{Ca});$$

$$I_{NaP} = \frac{I_{NaP,max}}{1 + (K_{m,Na}/[Na^+]_{in})^{1.5}}; I_{CaP} = \frac{I_{CaP,max}[Ca^{2+}]_{in}}{[Ca^{2+}]_{in} + K_{m,CaP}}; I_{Na} = g_{Na}m^3h(v_{DA} - E_{Na});$$

$$I_{Ca,T} = g_{Ca,T}rw(v_{DA} - E_{Ca}), I_{Ca,N} = g_{Ca,N}uf_{Ca,N}(v_{DA} - E_{Ca}); I_{Ca,L} = g_{Ca,L}pf_{Ca,L}(v_{DA} - E_{Ca});$$

$$I_{KDR} = g_{KDR}n(v_{DA} - E_{Na}); I_A = g_A qs(v_{DA} - E_K); I_{K,SK} = \frac{g_{K,SK}(v_{DA} - E_K)}{1 + (K_{M,SK}/[Ca^{2+}]_{in})^4},$$

$$m' = (\frac{1}{1 + e^{(-44.6 - v_{DA})/6}} - m)/\tau_m, h' = (\frac{1}{1 + e^{(-66.8 - v_{DA})/7.8}} - h)/\tau_h, q' = (\frac{1}{1 + e^{(-42 - v_{DA})/4}} - q)/\tau_q,$$

$$s' = (\frac{1}{1 + e^{(63 + v_{DA})/4}} - h)/50, n' = (\frac{1}{1 + e^{(-35 - v_{DA})/12}} - n)/10, r' = (\frac{1}{1 + e^{(-63.5 - v_{DA})/1.5}} - r)/\tau_r,$$

$$w' = (\frac{1}{1 + e^{(76.2 + v_{DA})/3}} - w)/\tau_w, u' = (\frac{1}{1 + e^{(-45 - v_{DA})/7}} - u)/\tau_u, p' = (\frac{1}{1 + e^{(-50 - v_{DA})/20}} - p)/\tau_p,$$

$$[Ca^{2+}]_{in}' = 2 \times f_{Ca}(I_{Ca,T} + I_{Ca,L} + I_{Ca,N} + I_{Ca,P} + I_{L,Ca})/(d_s f), [Na^+]_{in}' = 4 \times f_s(-I_{Na} - I_{Na,P} - I_{L,Na})/(d_s f).$$

Cortical neuron model

$$C_m {v_{cor}}' = -I_{leak} - I_{Na} - I_{Kd} - I_M - I_L + I_{appcor} - I_{th \to cor},$$

$$I_{leak} = g_l(v_{cor} - E_l); I_{Na} = g_{Na}m^3h(v_{cor} - E_{Na}); I_{Kd} = g_{Kd}n^4(v_{cor} - E_{Kd});$$

$$I_L = g_L q^2 r(v_{cor} - E_L); I_M = g_M p(v_{cor} - E_M),$$

$$m' = \alpha_m(v_{cor})(1 - m) - \beta_m(v_{cor})m, h' = \alpha_h(v_{cor})(1 - h) - \beta_h(v_{cor})h, n' = \alpha_n(v_{cor})(1 - n) - \beta_n(v_{cor})n,$$

$$q' = \alpha_q(v_{cor})(1 - q) - \beta_q(v_{cor})q, r' = \alpha_r(v_{cor})(1 - r) - \beta_r(v_{cor})r, p' = [p_\infty(v_{cor}) - p]/\tau_p(v_{cor}).$$

MSN neuron model

$$C_m {v_{msn}}' = -I_{NaF} - I_{NaP} - I_{KAf} - I_{KAs} - I_{KIR} - I_{KRP} - I_{BK} - I_{SK} - I_{CaL12} - I_{CaL13}$$
$$- I_{CaN} - I_{CaQ} - I_{CaR} - I_{CaT} - I(t) - I_{cor\to msn} - I_{DA\to msn},$$
$$I_{NaF} = g_{NaF}{m_1}^3 h_1(v_{msn} - E_{Na}); I_{NaP} = g_{NaP} m_2 h_2(v_{msn} - E_{Na}); I_{KAf} = g_{KAf}{m_3}^2 h_3(v_{msn} - E_K),$$
$$I_{KAs} = g_{KAs}{m_4}^2(a \times h_4 + (1-a))(v_{msn} - E_K); I_{KIR} = g_{KIR} m_5(v_{msn} - E_K);$$
$$I_{KRP} = g_{KRP} m_6(a \times h_6 + (1-a))(v_{msn} - E_K), a = 0.996;$$
$$I_{BK} = g_{BK}(v_{msn} - E_K), I_{SK} = g_{SK}(v_{msn} - E_K); I_{CaL12} = p_{CaL12}{m_7}^2(a \times h_7 + (1-a))H(V_{msn}), a = 0.17;$$
$$I_{CaL13} = p_{CaL13}{m_8}^2 h_8 H(V_{msn}); I_{CaN} = p_{CaN}{m_9}^2(a \times h_9 + (1-a))(V_{msn}), a = 0.21;$$
$$I_{CaQ} = p_{CaQ}{m_{10}}^2 H(V_{msn}); I_{CaR} = p_{CaR}{m_{11}}^3 h_{11} H(V_{msn}); I_{CaT} = p_{CaT}{m_{12}}^3 h_{12} H(V_{msn}),$$
$${m_i}' = [m_{i\infty}(v_{msn}) - m_i]/\tau_{m_i}(v_{msn}),$$
$${h_i}' = [h_{i\infty}(v_{msn}) - h_i]/\tau_{h_i}(v_{msn}).$$

Here i is from 1 to 12

$$H(V_m) = z^2 \frac{V_m F^2}{RT} \frac{[Ca^{2+}]_i - [Ca^{2+}]_0 \exp(-zFV_m/RT)}{1 - \exp(-zFV_m/RT)}$$

Table 1. Balance parameters in DA

parameters	values
f_s	4
f_{Ca}	0.005
d_s	$15\mu m$
F	$96520C/mol$

Table 2. Calcium parameters in MSN

parameters	values
z	2
F	$96489C/mol$
r	$8.31J/molK$
T	$35°C$
$[Ca^{2+}]_0$	$5mM$
$[Ca^{2+}]_i$	$0.001mM$

Table 3. Synaptic currents parameters

synaptic currents	$\bar{g}_{syn}(pS)$	$E_{syn}(mV)$	$\tau_{on}(ms)$	$\tau_{off}(ms)$
GABA	435	-80	0.25	3.75
AMPA	593	0	1.1	3.75
NMDA	600	0	2.81	160

Table 4. Synaptic currents parameters

Neuron	COR	DA	D1	D2	GPi	GPe	STN	TH
COR	-	-	NMDA:0.001	NMDA:0.0035	-	-	AMPA:0.1<br>NMDA:0.1	-
DA	-	-	AMPA:10.01	GABA:22	-	-	-	-
D1	-	-	-	-	GABA:30.6	-	-	-
D2	-	-	-	-	-	GABA:30	-	-
GPi	-	-	-	-	GABA:0.0001	-	-	GABA:0.5
GPe	-	-	-	-	GABA:30.5	GABA:0.1	GABA:0.1	-
STN	-	-	-	-	AMPA:0.0014<br>NMDA:0.0014	AMPA:0.11<br>NMDA:0.0	-	-
TH	NMDA:0.001	-	-	-	-	-	-	-
I_{app}	0.2	2	0	0	-0.65	-0.3	0	0.04
